# Spinal Epidural Lipoma on the Ventral Dura Side and Intervertebral Foramen Causing Lumbar Radiculopathy

**DOI:** 10.1155/2022/7502552

**Published:** 2022-10-27

**Authors:** Hiroshi Noguchi, Masao Koda, Tetsuya Abe, Toru Funayama, Hiroshi Takahashi, Kousei Miura, Kentaro Mataki, Mamoru Kono, Fumihiko Eto, Yosuke Shibao, Masashi Yamazaki

**Affiliations:** Department of Orthopaedic Surgery, Faculty of Medicine, University of Tsukuba, 1-1-1 Tennodai, Ibaraki 305-8575, Japan

## Abstract

A 56-year-old obese man with a body mass index of 30.9 kg/m^2^ presented with left sciatica and intermittent claudication. Computed tomography scans showed a posterior vertebral scalloping change in L3, L4, and L5. Meanwhile, magnetic resonance imaging revealed epidural mass posterior to the L3, L4, and L5 vertebral bodies. The solitary mass was isosignal to subcutaneous fat and asymmetrically compressed to the left side of the dural sac and L4 nerve root, as observed on axial T1- and T2-weighted images. To the best of our knowledge, there have been few reports of a solitary epidural lipoma causing lumbar radiculopathy. The patient underwent transforaminal lumbar interbody fusion at L4–L5, and his symptoms then resolved. Thus, we recommend decompression and fixation as appropriate management for lumbar radiculopathy caused by epidural lipoma located on the ventral side of the dura and intervertebral foramen.

## 1. Introduction

Spinal epidural lipomatosis (SEL), the abnormal accumulation of unencapsulated adipose tissue in the epidural space of the spinal canal, is a well-known rare condition with distinctive magnetic resonance imaging (MRI) findings [1, 2]. Moreover, intraspinal extradural lipomas are rare, accounting for only 0.2%–0.8% of all intraspinal tumors [3–6]. Radiculopathy is commonly due to herniated discs and spinal canal stenosis, although the possibility of lipomatous tissue compression should also be considered.

Here, we present the clinical, radiographic, and pathological assessment of a patient with a rare case of lumbar radiculopathy and intermittent claudication secondary to an epidural lipoma located on the ventral side of the dura and intervertebral foramen.

## 2. Case Report

### 2.1. History and Examination

A 56-year-old man presented with left anterior thigh pain, left sciatica, and intermittent claudication for 5 years, and his symptoms had worsened within 1 year. The patient had no history of corticosteroid use or endocrinopathy.

**Figure 1:**
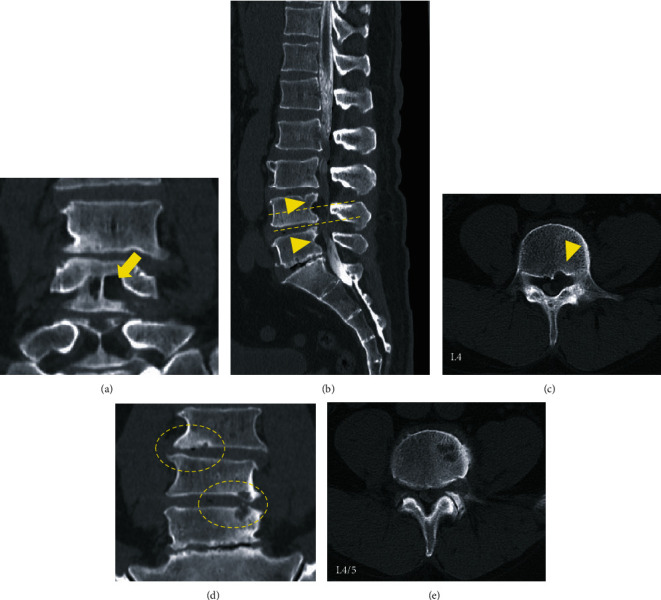
Computed tomography scans in coronal (a, d), sagittal (b), and axial (c, e) views showing posterior vertebral scalloping changes of L3, L4, and L5 without erosion of the cortical and medullary bone (arrowheads) and the cavity of the posterior wall connecting the foramen (arrow). Schmorl's nodes at the endplates of L3, L4, and L5 are observed (circle).

**Figure 2:**
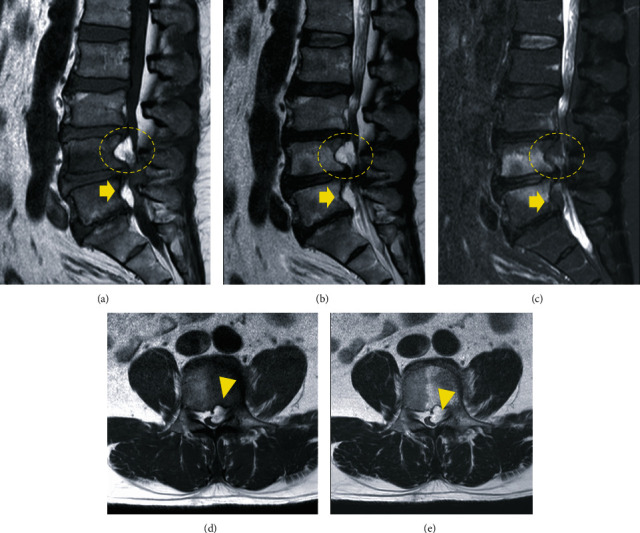
Magnetic resonance imaging on T1-weighted (a), T2-weighted (b), and T2-fat suppression (c) reveal a mass (circles) of high-signal intensity at the L4 pedicle level in the left epidural space. The following were observed: a hyperintense anterior epidural mass (arrows) with strangulation of the dural sac at L4-5 levels (the first row), epidural mass (arrowheads) at L4 compressed asymmetrically on the left side of the cauda equina, and exiting left L4 nerve root on axial T1- and T2-weighted images (d, e) (the second row).

**Figure 3:**
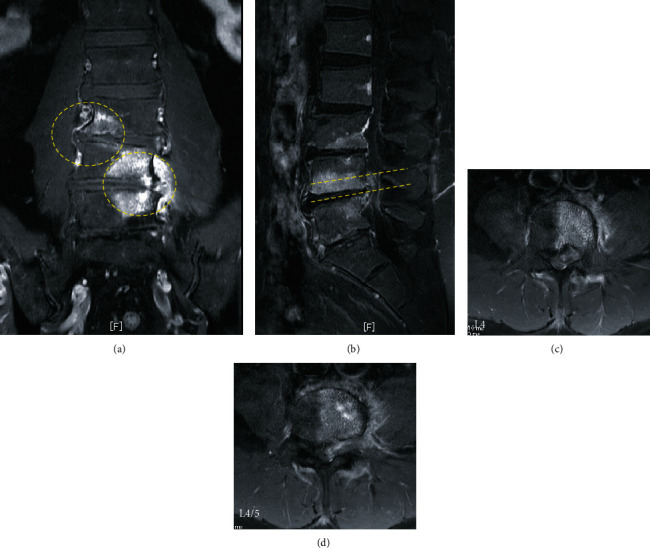
Contrast-enhanced MRI scans in coronal (a), sagittal (b), and axial (c, d) views reveal an epidural mass at L4/5 and bone defects with concentric rim of edema (circle) (hypointense in T1-weighted, hyperintense in T2-weighted and short tau inversion recovery (STIR)) in the endplates of the L3, L4, and L5 vertebrae and acute Schmorl's nodes.

On admission, he was obese (height, 180 cm; weight, 100 kg; body mass index, 30.9 kg/m^2^), the extension of the lumbar spine was limited, and the Kemp sign was positive on the left side. The patient had severe limitations in the left lower limb in performing straight leg raise test, although no motor weakness or hypoesthesia was elicited in further tests. Plain radiographs showed no abnormality, instability, or other pathologic findings. Computed tomography (CT) revealed posterior vertebral scalloping change of L3, L4, and L5 without erosion of the cortical and medullary bone and Schmorl's nodes at the endplate of the L3, L4, and L5 (Figure 1). Meanwhile, MRI revealed an epidural mass posterior to the L3, L4, and L5 vertebral bodies with isosignal intensity to subcutaneous fat and vertebral scalloping. We observed a hyperintense epidural mass at L4, which asymmetrically compressed the left side of the dural sac and left L4 nerve root on axial T1- and T2-weighted MRI (Figure 2) and gadolinium contrast-enhanced imaging (Figure 3). Thus, a spinal epidural lipoma was suspected with differential diagnoses of epidural tumor and neurinoma.

### 2.2. Surgery

**Figure 4:**
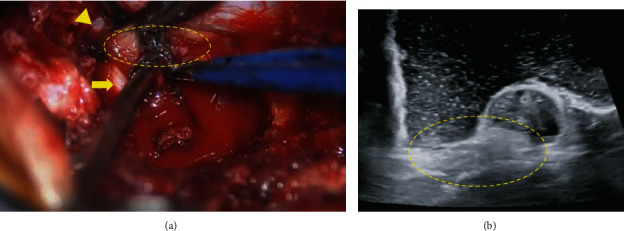
(a) Adipose tissue mass (circle) between the left L4 nerve root (arrow) and dural sac (arrowhead) intraoperatively. (b) Intraoperative echo image reveals the location of adipose tissue mass (circle) ventral to the dura, with no invasion to the intradural space.

We recommended surgery because the patient's leg pain and sciatica were only slightly responsive to pain killers for half a year and L4 nerve root block. To directly observe the mass spreading to the intervertebral foramen, we performed a transforaminal lumbar interbody fusion of L4–5 and removed the facet joint. Intraoperatively, a well-demarcated, yellowish-white encapsulated adipose tissue mass was found in the ventral side of the dura advancing to the hollow of the posterior wall. Moreover, the lipomatous mass was present along the left L4 nerve root (Figure 4) and tightly adhered to the epidural venous plexus and ventral dura mater; thus, it could not be easily detached. After mass dissection, stenosis of the dural sac at the level of L4–5 and compression of the left L4 nerve root were released.

### 2.3. Histological Analysis and Postoperative Course

**Figure 5:**
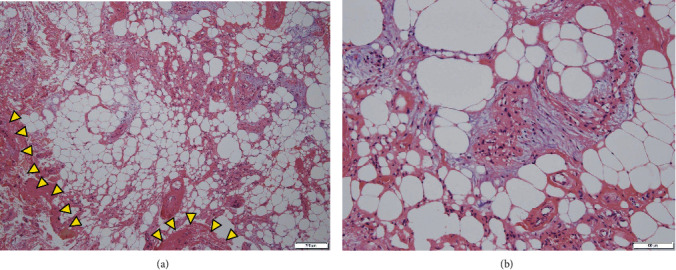
Hematoxylin and eosin-stained microscopic findings ((a) ×40 and (b) ×100) confirm mature adipose tissue with large adipocytes enclosed by fibrous tissue (arrowheads), compatible with a lipoma.

Histological examination of the encapsulated epidural mass showed lobulated mature adipose tissue enclosed by fibrous tissue, compatible with a lipoma (Figure 5). Inflammatory cell infiltration was observed in the lipoma, but no vascular atypia in the final histopathology results. One month after surgery, the patient reported significant pain relief in the left leg, allowing him to walk smoothly. One year after surgery, there was no recurrence of sciatica and intermittent claudication; hence, the patient was satisfied with the management.

## 3. Discussion

Both SEL and epidural lipoma are rare disorders characterized by abnormal adipose tissue proliferation in the extradural space, compressing the neural elements. In both conditions, accompanying symptoms of cauda equina syndrome and radiculopathy depend on the size and location of the tumor. Therefore, it is difficult to distinguish between SEL and epidural lipoma based on symptoms alone.

The process of differentiating these lipomatous lesions starts with a survey of the following SEL risk factors: obesity, chronic exogenous steroid use, and endocrinopathy. Loriaux et al. reported that most patients with intraforaminal lipoma presented with none of the characteristics associated with SEL [5]. However, our patient presented with an established SEL risk factor: obesity.

In addition to radiographic examination, CT can be used to assess the lipomatous lesion and bony architecture, such as scalloping of the posterior vertebral wall [2]. Moreover, MRI allows diagnosis of epidural lipoma and differentiation from SEL, which has specific findings of a hyperintense (T1-weighted) and intermediate intensity (T2-weighted) epidural mass, ruling out epidural hematoma, abscess, or tumor. Furthermore, SEL typically occurs around the spinal dura in the lumbar spinal canal space and shows Y-shaped signs of lumbar thecal compression on axial MRI [1, 2]. In our case, the enhancement of the lipomatous epidural tissue was evident on the ventral side of the spinal dura and in the foramen. These points differ from a typical SEL, and they are characteristic imaging findings that should be suspected of lipoma.

Intraoperative gross and pathological findings are valuable in differentiating between SELs and lipomas. The macroscopic characteristic of SEL is a noncapsulated epidural mass caused by hyperplasia of normal epidural fat [2, 4]. In contrast, an epidural lipoma is encapsulated by fibrous tissue, with microscopic findings of mature adipose tissue [3–6]. In our case, unlike typical lipomas, fibrotic tissue was abundantly mixed, and some inflammatory cells were also found.

Reported conservative approaches including weight loss interventions or steroid medication dose reduction may be adopted to reduce the risk of SEL [1]. Moreover, selective nerve root block can relieve severe symptoms caused by epidural lipoma-induced radiculopathy. In cases of conservative management failure or more severe clinical symptoms, surgery may be indicated. Laminectomy with the removal of epidural adipose tissue is the mainstream procedure [2–6], which has a generally uneventful and good postoperative course. In our case, the extradural lipoma was present not only in the spinal canal but also in the intervertebral foramen, making it difficult to secure the visual field without excising the facet joints. Consequently, transforaminal lumbar interbody fusion with intervertebral arthrotomy appeared to be an easier and safer method than working around the dura mater and nerve roots after laminectomy to examine lipomatous lesions that were present on the ventral side of the dura mater or along the nerve root.

**Figure 6:**
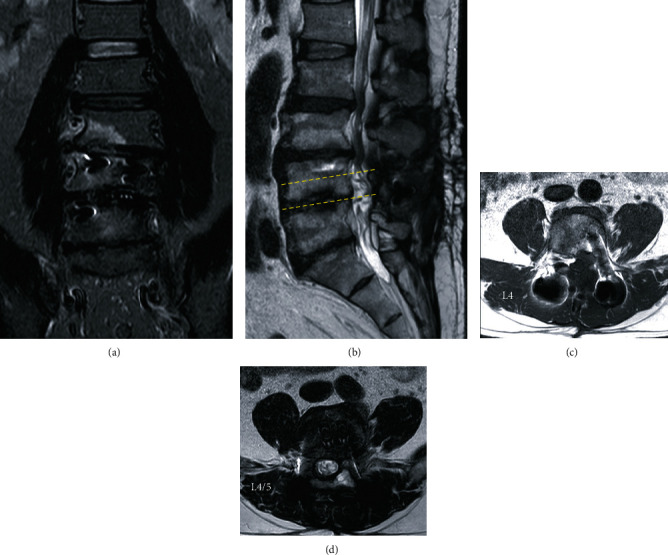
Magnetic resonance imaging on short tau inversion recovery (a), T2-weighted (b, d), and T1-weighted (c) at 1 year after surgery. The epidural lipoma was completely resected while the dural canal and nerve roots were decompressed. The endplate degeneration finding at the L4/5 level was improved.

MRI showed obvious intensity changes in the vertebral body. The degenerative marrow changes (Modic type 1) and Schmorl's nodes at the endplates of the L3, L4, and L5 vertebrae were demonstrated with SEL in our case. Although endplate degeneration findings (Modic type 1) in patients with chronic low back pain have been suggested to be associated with a high rate of low back pain [7], the patient in this case had only mild low back pain without exacerbation during the current episode. In addition, the patient was not aware of any low back pain, although the same initial findings remained at the L3/4 level on MRI 1 year after surgery (Figure 6). Thus, we believe that these degenerative changes were not directly related to his complaints relating to the current study.

However, these changes may affect radiculopathy. Kim et al. reported a case of solitary epidural lipoma causing lumbar radiculopathy associated with unilateral arthritis of the posterior facet joint [6]. They presumed that any instability may make the nerve root more vulnerable to compression due to lipoma, and this instability may be a causative factor for abrupt symptom onset. In our case, the degenerative endplate changes of the L4−5 may cause abnormal movements between spinal segments and affect inflammatory reactions around adipose tissue. One year later, MRI showed that the signal change at the L4/5 level was improved by intervertebral fusion (Figure 6).

Intraspinal epidural lipomas are very rare and may be easily overlooked because of their subtle MRI findings that are similar to those of normal anatomical variations or SEL. This case highlights the importance of considering an epidural lipoma when MRI reveals an epidural compressive and asymmetric fatty encapsulated lesion.

In conclusion, although MRI can diagnose an epidural lipomatous mass, additional procedures may be required to examine the mass. We recommend transforaminal lumbar interbody fusion for the inspection of an epidural lipoma, especially when it is located on the ventral side of the dura and intervertebral foramen.

## Data Availability

The datasets used in this study are available from the corresponding authors on reasonable request.
